# Continual Antigenic Diversification in China Leads to Global Antigenic Complexity of Avian Influenza H5N1 Viruses

**DOI:** 10.1038/srep43566

**Published:** 2017-03-06

**Authors:** Yousong Peng, Xiaodan Li, Hongbo Zhou, Aiping Wu, Libo Dong, Ye Zhang, Rongbao Gao, Hong Bo, Lei Yang, Dayan Wang, Xian Lin, Meilin Jin, Yuelong Shu, Taijiao Jiang

**Affiliations:** 1 College of Biology, Human University, Changsha, 410082, China; 2 Institute of Biophysics, Chinese Academy of Sciences, Beijing, 100101, China; 3 National Institute for Viral Disease Control and Prevention, China CDC, Beijing, 100052, China; 4 College of Animal Science & Medicine, Huazhong Agricultural University, Wuhan, 430070, China; 5 Center of System Medicine, Institute of Basic Medical Sciences, Chinese Academy of Medical Sciences & Peking Union Medical College, Beijing, 100005, China; 6 Suzhou Institute of Systems Medicine, Suzhou, Jiangsu, 215123, China

## Abstract

The highly pathogenic avian influenza (HPAI) H5N1 virus poses a significant potential threat to human society due to its wide spread and rapid evolution. In this study, we present a comprehensive antigenic map for HPAI H5N1 viruses including 218 newly sequenced isolates from diverse regions of mainland China, by computationally separating almost all HPAI H5N1 viruses into 15 major antigenic clusters (ACs) based on their hemagglutinin sequences. Phylogenetic analysis showed that 12 of these 15 ACs originated in China in a divergent pattern. Further analysis of the dissemination of HPAI H5N1 virus in China identified that the virus’s geographic expansion was co-incident with a significant divergence in antigenicity. Moreover, this antigenic diversification leads to global antigenic complexity, as typified by the recent HPAI H5N1 spread, showing extensive co-circulation and local persistence. This analysis has highlighted the challenge in H5N1 prevention and control that requires different planning strategies even inside China.

The highly pathogenic avian influenza (HPAI) H5N1 virus has become of global concern since the isolation and identification of the strain A/Goose/Guangdong/1/1996 (GsGD) in Guangdong province of China in 1996^1^,^2^,^3^. Since then, the GsGD lineage of HPAI H5N1 virus has spread into many countries and regions in Asia, Europe, Africa and North America, causing epizootic and panzootic infections in birds of many species, killing tens of millions of birds and spurring the culling of hundreds of millions of poultry to halt its spread^1^,^3^. Moreover, as of 21 November 2016, sporadic infections of the HPAI H5N1 virus have been responsible for 452 known fatalities among 856 confirmed human infections^4^.

Given the wide spread of the virus among animals of many species and a relatively high fatality rate in humans following zoonotic infection, the concern of a host jump that would allow human-to-human spread has led to global efforts to prepare for a potential devastating threat^5^,^6^. However, due to its propagation in multiple hosts, diverse H5N1 viral populations exist that comprise of genetic variants, shaped by collected mutations and frequent re-assortments of genes from different strains^7^,^8^,^9^,^10^,^11^. This diversity is higher than that observed for seasonal influenza viruses like human H1N1 and H3N2^12^,^13^. Given this diversity, H5N1 antigenic variants can rapidly evolve to escape host immune surveillance. Moreover, the dissemination of the virus is complicated. Previous studies have shown that the global persistence of the HPAI H5N1 virus results from the interplay between a high capacity to persist in domestic poultry in localized areas, combined with sporadic long-distance introduction events involving migratory birds^1^,^14^,^15^. This makes the battle against the HPAI H5N1 virus quite a challenge.

Because vaccination is currently the most effective way to prevent and control infections by influenza viruses, several variants of the HPAI H5N1 virus have been recommended as vaccine strains for protection of poultry^3^,^16^, and it has been proposed that such vaccines should be stockpiled to be prepared for future outbreaks^3^,^17^. However, due to the rapid evolution of the virus and its unknown evolutionary patterns, in many cases vaccines for poultry are not well matched to the strains in circulation, and such vaccines could actually drive the evolution of the virus^18^,^19^,^20^,^21^. Therefore, understanding the evolution of HPAI H5N1, especially the evolution of its antigenicity in a temporal-spatial manner, is critical for efficient prevention and control of the virus. Despite multiple global efforts, the antigenic evolution of HPAI H5N1 is not adequately understood^22^,^23^.

For ease of tracking the evolution of the virus, the H5N1 Evolution Working Group (HEWG), a joint effort of the World Health Organization (WHO), World Organisation for Animal Health (OIE) and Food and Agriculture Organization (FAO), has designed a nomenclature to classify the GsGD lineage of Eurasian HPAI H5N1 viruses^24^,^25^, based on the phylogeny of the antigen hemagglutinin (HA). According to this nomenclature, all viruses of the GsGD lineage are classified into 10 clades (numbered 0 to 9), which are further subdivided into second-order, third-order and even fourth-order subclades. Although this system is very comprehensive, it is more reflective of the genetic than of the antigenic properties of the virus. For example, based on the cross-reactivity to a panel of 17 monoclonal antibodies raised against HPAI H5N1 strains, Wu and colleagues found that the seven recognized genetic clades of HPAI H5N1 (isolated between 2002 and 2007 in Asia) could actually be grouped into four distinct antigenic groups^26^. Antigenic grouping of virus strains would facilitate the recognition of emerging antigenic variants, thus aiding to the selection of vaccine strains^27^. Moreover, due to the rapid evolution of the virus, the classification of HPAI virus based on phylogenic analysis will become very complicated and hard to interpret over time.

China, especially southern China, is often quoted as the source for the HPAI H5N1 virus, owing to its complex ecology and diverse geographical features^15^,^28^,^29^. However, influenza virus antigenic evolution in China and its impact on global influenza dynamics is not adequately understood, in part due to a lack of sufficient viral data originating from China. In this study, we carried out the large-scale sequencing of the HA genes of 218 HPAI H5N1 viruses, isolated from representative regions of mainland China. Through accurate modeling of these newly sequenced virus and viruses with HA gene sequences available from public databases, we developed a comprehensive picture of the antigenic evolution of HPAI H5N1 viruses across the globe.

## Results

### Sequencing of HPAI H5N1 viruses from mainland China

**Figure 1:**
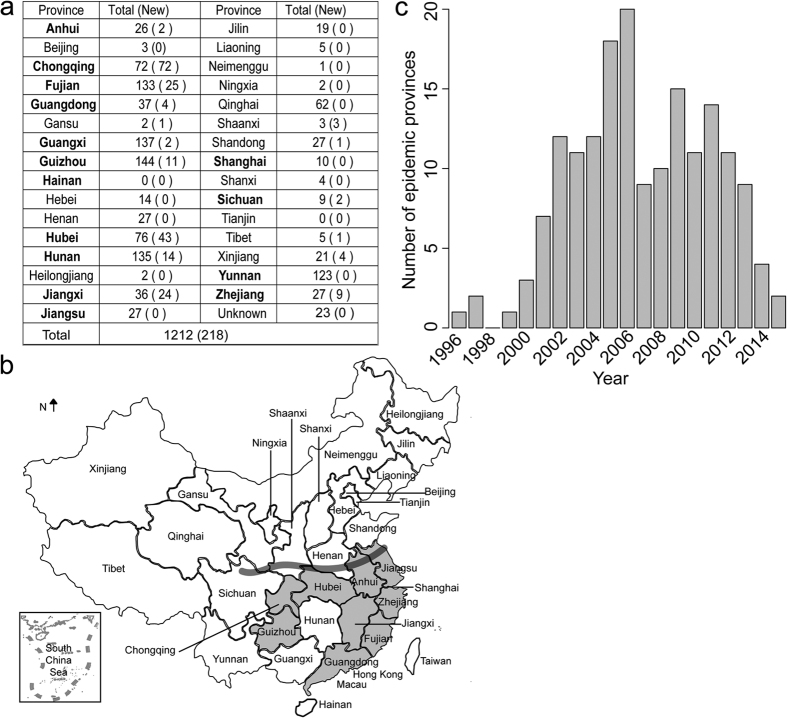
The sequencing of 218 HPAI H5N1 viruses sampled from mainland China. (**a**) Number of HA sequences for HPAI H5N1 virus sampled per province of mainland China. The number in the brackets refers to the number of newly sequenced viruses. Provinces in southern China are in bold text. (**b**) A map of China showing the all provinces, and, in grey, provinces in which epidemics caused by HPAI H5N1 virus were identified in the year 2013. The heavy gray line indicates the position of the Qin Mountains and Huai River that divide northern and southern China. The map was reconstructed using OpenStreetMap (http://www.openstreetmap.org/), licensed on terms of the Open Database License, “ODbL” 1.0 (http://opendatacommons.org/licenses/odbl/), and is for illustrative purposes only. (**c**) The number of provinces with epidemics caused by HPAI H5N1 virus inferred from HA sequences in mainland China from 1996 to 2015.

To obtain a better understanding of the evolution of HPAI H5N1, we sequenced HA of 218 HPAI H5N1 viruses isolated from mainland China (Fig. 1a and Supplementary Table S1). These newly derived sequences constituted 18% of the sequences from mainland China available until now (Fig. 1a). Most of the sequences represent isolates from 2006 onwards, due to the difficulty to derive virus samples from earlier years. From a geographical point of view, the areas we sampled the viruses covered 16 provinces (Fig. 1a), which represent nearly half of the provinces of the country. Important to note is that, except for Hainan, Jiangsu, Shanghai and Yunnan, the sampling covered all provinces of southern China (area south of the heavy gray line in Fig. 1b), a region supposed to be the epicenter of the HPAI H5N1 virus^15^,^28^,^29^. In addition, a number of samples were derived from provinces in northern China (north of the heavy gray line in Fig. 1b) which experienced human infections or frequent H5N1 epidemics, such as Xinjiang, Shaanxi, Shandong provinces. For provinces Chongqing, Hubei, Jiangxi and Shaanxi, the newly derived sequences outnumber those available in the public database. Complementing the newly derived sequences with sequences available in the public databases allowed for an in-depth investigation on the evolutionary dynamics of HPAI H5N1 virus in China.

The time distribution of the sequenced isolates roughly reflected the epidemic dynamics of HPAI H5N1 in mainland China (Fig. 1c). In earlier years from 1996–1999, HPAI H5N1 virus only caused sporadic epidemics in a few provinces in southern China, such as Guangdong and Guangxi^15^. The virus became more prevalent in poultry and wild birds in mainland China after the year 2000, as shown by the increasing number of provinces experiencing epidemics. In 2005 and 2006, widespread outbreaks occurred in mainland China. In these two years, more than 15 provinces reported HPAI H5N1 epidemics, inferred from the sequences available. Although the number of provinces suffering from H5N1 epidemics decreased strongly after 2006, the virus was not eliminated: between 2007 and 2015, there were on average nine provinces with H5N1 outbreaks each year (Fig. 1c). The situation in 2013, during which nine provinces experienced H5N1 epidemics, exemplified by the grey color in Fig. 1b, showing all were located in southern China.

### High-confidence modeling of antigenic clusters of HPAI H5N1 viruses

**Figure 2:**
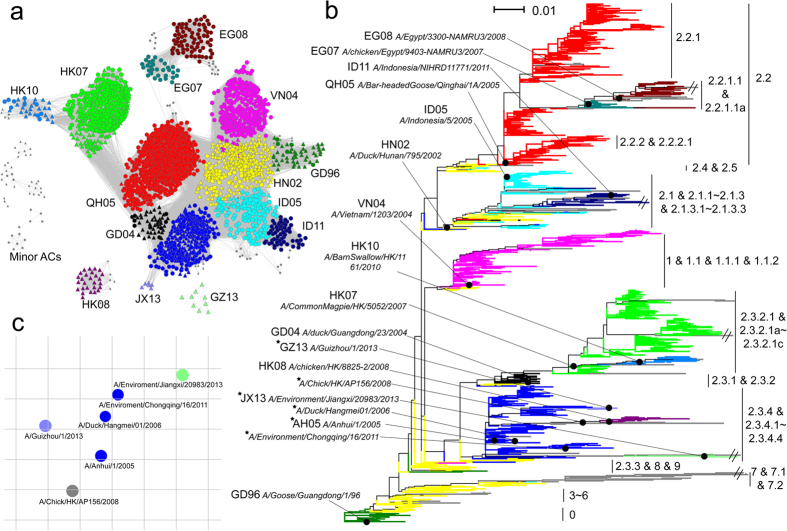
High-confidence modeling of ACs of HPAI H5N1 viruses. (**a**) Predicted antigenic correlation network (ACnet) of the ACs defined for 2441 HPAI H5N1 viruses with unique HA1 protein sequences. All pairs of viruses which were predicted to be antigenically similar were connected in ACnet. Triangles in the network refer to the viruses from China. The names for the major ACs (in color) are indicated, while minor ACs are shown in gray. (**b**) Phylogenetic tree of the 2441 HA1 sequences, colored according to the predicted ACs. The sub-clades to which the viruses belong (H5N1 Evolution Working Group nomenclature) are shown to the right. The branch length was scaled according to the legend in the top left. The strains listed to the left of the tree refer to the strains used for naming the predicted major ACs. The stars indicate strains used in the HI assay. (**c**) The antigenic cartography for six representative viruses of four antigenic clusters which were mainly composed of viruses of clade 2.3.4 and its sub-clades. The viruses were colored according to the antigenic clusters they belong to.

We developed an antigenic modeling tool, which we called PREDAC-H5-C, to divide viruses into antigenic clusters (AC), based on the immunogenic part of HA protein sequences (HA1, see Methods). The PREDAC-H5-C tool facilitated a systematic investigation of the antigenic evolution of all investigated HPAI H5N1 viruses. To determine which clustering most accurately reflected the actual antigenic evolution, an antigenic dataset including 798 pairs of HPAI H5N1 viruses with known antigenic relationship (see Methods) was constructed. It should be noted that none of the viruses in the antigenic dataset had been used in training the PREDAC-H5-C model. The rationale behind this was that if the antigenic clustering accurately captured the actual antigenic relationship of HPAI H5N1 viruses, the viruses within predicted ACs should be antigenically similar, while viruses grouped in different ACs should differ more in antigenicity (Supplementary Methods). Thus, with the help the antigenic dataset, the best antigenic clustering by PREDAC-H5-C was obtained, which achieved an agreement of 0.81 with the antigenic dataset (Supplementary Fig. S1). It separated a total of 5605 viruses, including 218 newly sequenced isolates and 5387 sequences collected from public databases, into 36 ACs (Fig. 2a and Supplementary Table S2). Among them, 15 major ACs were defined which covered 97% of all viruses analyzed. They were named after their representative viruses (in most cases the WHO-recommended vaccine strain), by two letters that refer to the country/region of isolation followed by two digits that refer to the year of isolation. For example, the AC GD96 was named after the strain A/Goose/Guangdong/1/1996 (Fig. 2). Besides for the major ACs, the other ACs were defined as minor ACs (see Methods).

When mapping the predicted ACs to the phylogenetic tree of these viruses (comparing Fig. 2a and b), we found that a predicted AC generally covered a side lineage or comprised several closely related side lineages. We further tried to compare the predicted ACs with clade IDs as per the nomenclature system of the HEWG, but since the designated clades for H5N1 are hierarchically organized, this was not straightforward. Table S2 summarizes the correspondence between our predicted ACs and the designated clades or sub-clades. Overall, a good correspondence was observed: a pair of viruses had a probability of 0.94 to be found in the same predicted AC if they were from the same phylogenetic clade/sub-clade, and that probability was 0.90 for strains to be divided in different ACs if they belonged to different phylogentic clades/sub-clades. We further assessed how well the predicted ACs and designated HEWG clades matched with the antigenic data. The ratio of antigenically similar pairs within a predicted AC was similar to that based on HEWG clades (0.78), while the ratio of antigenically different pairs between the predicted ACs was much larger than that based on HEWG clades (0.85 versus 0.73). This suggests the predicted ACs are more accurate in describing antigenic relationships than the HEWG nomenclature system. This can be exemplified with HEWG clade 2.2, as shown in Fig. 2b. The clade is divided into tertiary sub-clades 2.2.1 and 2.2.2 and further into quaternary clade 2.2.1.1, but most of these viruses fell into our predicted AC QH05 (marked in red) except for members of the quaternary sub-clade 2.2.1.1. Experimental data confirmed that indeed only the quaternary 2.2.1.1 was antigenically different from the other viruses in clade 2.2, while the clades 2.2.1 and 2.2.2 contained viruses that are antigenically similar to clade 2.2 (Supplementary Table S3). Interestingly, the quaternary clade 2.2.1.1 comprises two predicted ACs, EG07 and EG08, which were also reported to be antigenically different (Supplementary Table S3).

To further demonstrate the accuracy of the predicted ACs, the antigenic relationship between viruses of clade 2.3.4 and its sub-clades 2.3.4.1 to 2.3.4.4, which circulated most extensively in mainland China in recent years, were determined with the HA-inhibition (HI) assay. The viruses belonged to 8 ACs (Supplementary Table S2); of these, four (AH05, GZ13, JX13 and Minor-28) were included in the assay, as shown in Fig. 2c (also see Supplementary Table S4). Although some antigenic heterogeneity was observed in AH05, these four ACs were antigenically all distinguishable with each other by the HI assay, confirming that by and large the predicted ACs reflect true antigenic relationships.

### The origins of ACs of HPAI H5N1 viruses

**Figure 3:**
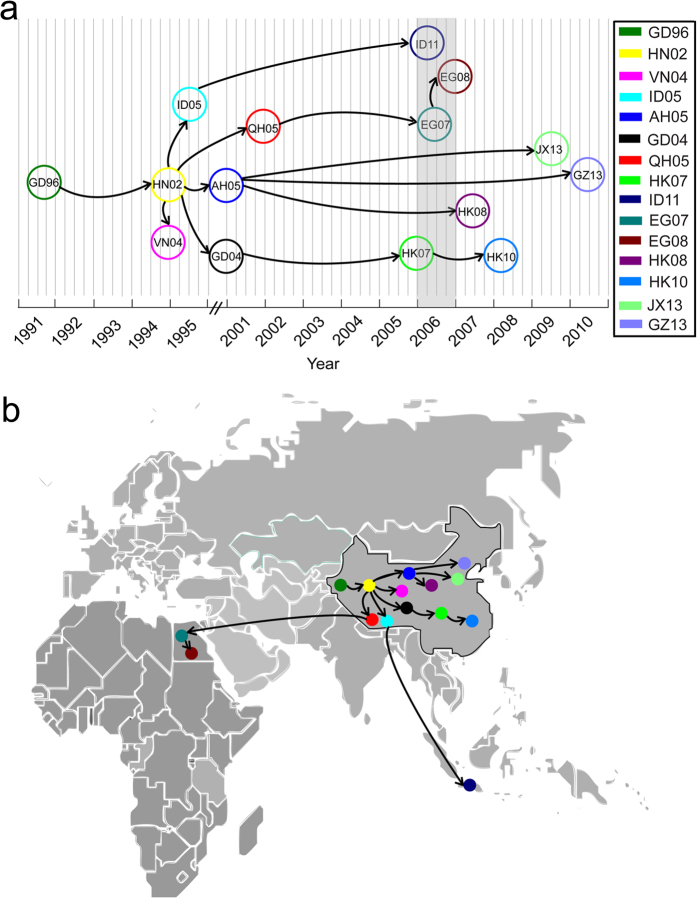
The origins and evolutionary pathways of predicted major ACs of HPAI H5N1 virus. (**a**) The tMRCA for major ACs (encircled) and their phylogenetic relationship, as inferred from Fig. 2b. (**b**) The most probable source country for each major AC. The map was reconstructed using OpenStreetMap (http://www.openstreetmap.org/), licensed on terms of the Open Database License, “ODbL” 1.0 (http://opendatacommons.org/licenses/odbl/), and is for illustrative purposes only.

To find out how the predicted ACs had originated, we dated the timing of their most recent common ancestors (tMRCA) and inferred the most probable source countries for the major ACs (Supplementary Table S5). As summarized in Fig. 3a, although the first strain of GsGD lineage was isolated in 1996 in the Guangdong province of China, the emergence of the first AC GD96 could be dated back to the end of 1991. Then GD96 gave rise to HN02 in the beginning of 1995. The HN02 had been hidden for years until its discovery in 2000. Remarkably, HN02 had five propagations. Two (VN04 and ID05) were generated right after HN02 appeared, while the other three (QH05, AH05, and GD04) were generated around 2001–2002. Interestingly, there was a burst of generation of four new ACs around 2006, when widespread outbreaks of HPAI H5N1 viruses were observed in China and Southeast Asia. The latest two ACs (JX13 and GZ13) were generated around 2010. We further mapped the source countries of these predicted major ACs (Fig. 3b). Overall, 12 of the 15 major ACs were generated in China, while ID11 originated in Indonesia and EG07 and EG08 evolved in Egypt.

### Antigenic diversification with the spread of the virus in China

**Figure 4:**
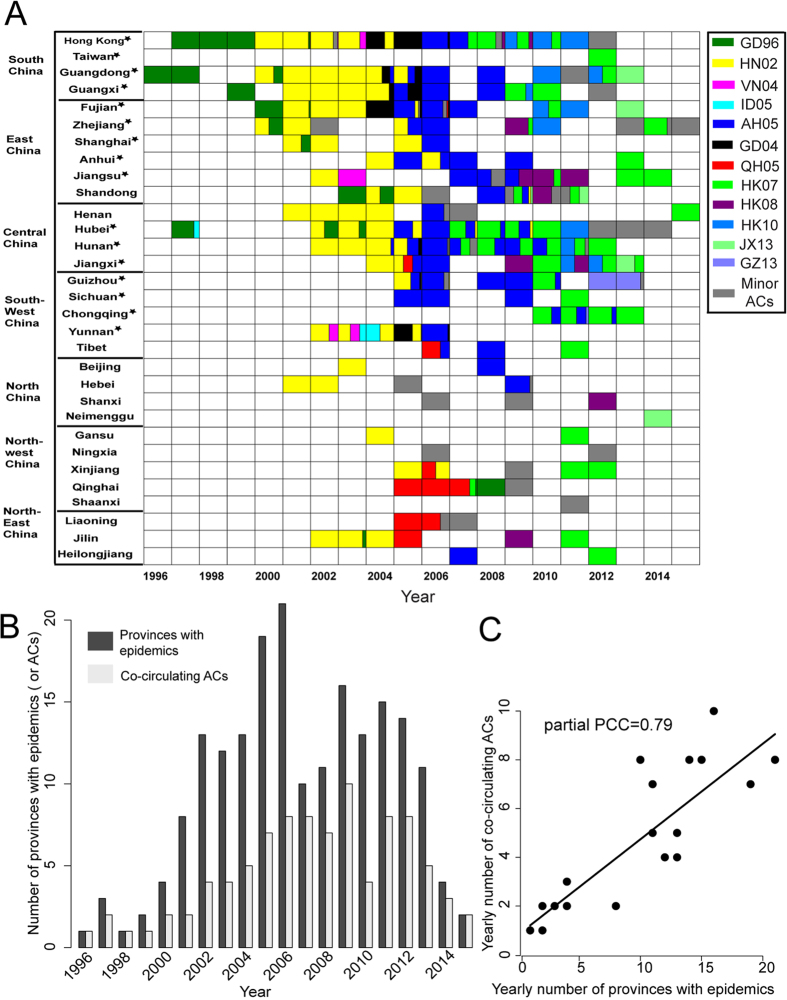
Translocation of antigenic types of HPAI H5N1 virus in China. (**A**) Tempo-spatial dynamics of ACs within China, showing dynamic changes in the fraction of ACs (colored as in Fig. 2) recorded on a yearly basis for each province. White indicates no virus is isolated in that province in that year. (**B**) The number of provinces with epidemics (dark gray) and co-circulating ACs (light gray) between 1996 and 2015. (**C**) The partial Pearson Correlation Coefficient (PCC) indicating the correlation between the number of province with epidemics and co-ciruclating ACs after controlling for the influence of the number of HA sequences per year.

We sought to investigate in detail the antigenic evolution of the virus in China. Figure 4A shows the overall evolutionary dynamics of the predicted ACs in China (here combining mainland China with Hong Kong) from 1996 to 2015. In the early years during 1996–1999, HPAI H5N1 virus consistently belonged to AC GD96 and mainly circulated in South China (including Hong Kong). In 2000, GD96 was replaced by the predicted AC HN02 which spread rapidly across the country. HN02 continued to dominate until 2004. During 2005–2009, AH05 became predominant and in 2010 HK07 became predominant. Also note that besides the dominant ACs, quite a few major and minor ACs co-circulated which added up to a significant portion in each year since 2002; they could actually dominate in some provinces. For example, in 2006, although the dominant AC AH05 occupied almost the whole southern part of China, the major AC QH05 and a few minor ACs became dominant in northern China. This exemplifies the antigenic complexity of HPAI H5N1 virus populations in the country. Panels b and c of Fig. 4 clearly show that the antigenic diversification of the virus was co-incident with its geographic expansion in China. We observed a significant correlation between the number of predicted ACs in circulation and the number of provinces in epidemic inferred from the HA-sequenced viruses, with a partial Pearson Correlation Coefficient (PCC) of 0.79 (p-value 6.9e-8). On a global scale, however, there was no significant correlation between the number of predicted ACs in circulation and the number of countries with epidemics inferred from the HA-sequenced viruses (data not shown).

### Global co-circulation of ACs of recent H5N1 viruses

**Figure 5:**
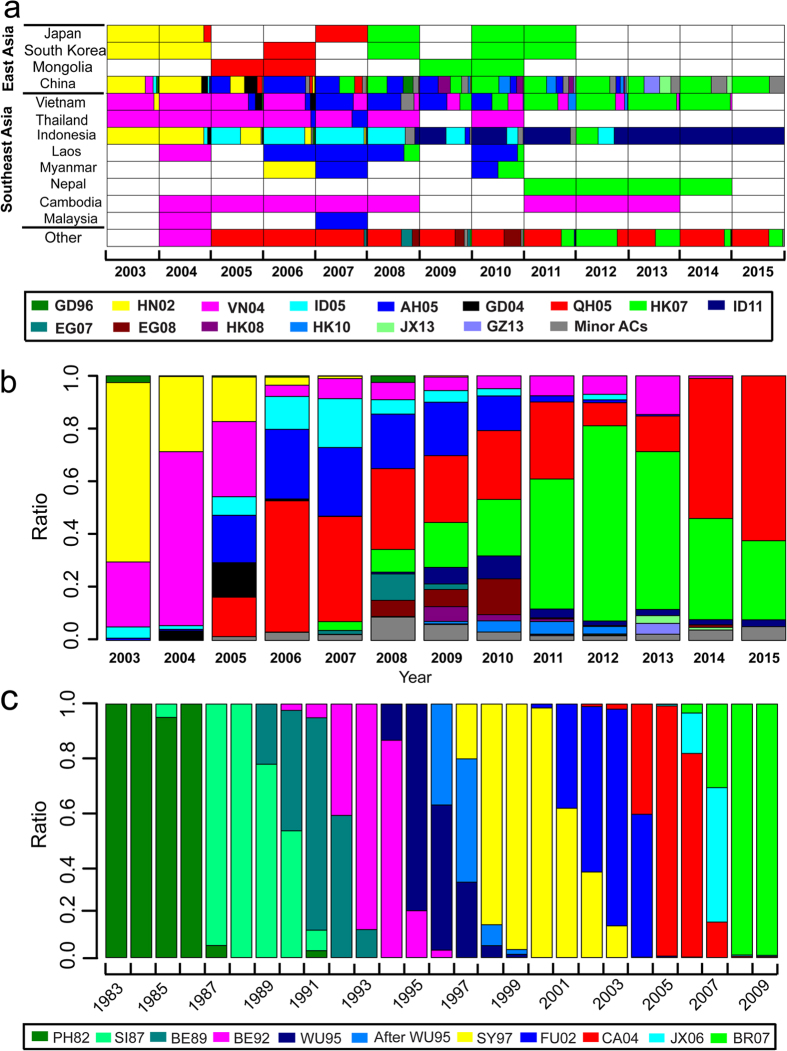
Global co-circulation of multiple ACs of recent H5N1 viruses compared to the seasonal human influenza H3N2 virus. (**a**) Global tempo-spatial dynamics of HPAI H5N1 ACs from 2003 to 2015 (for details see Supplementary Fig. S2). Coloring is similar as in Fig. 4. (**b**) The yearly global proportion of ACs of HPAI H5N1 virus from 2003 to 2015. (**c**) The yearly global composition of ACs of human influenza H3N2 virus, covering the years 1983 to 2009 (adapted from Du’s work^27^).

As demonstrated above, since the detection/discovery of the first HPAI H5N1 virus in South China in 1996, its antigenic types have altered dramatically, not only in China but also across the globe. Therefore, we further investigated the antigenic evolution of recent H5N1 viruses across the globe. As shown in Fig. 5a, the antigenic diversity in East and Southeast Asia is much larger than that in other regions, including Europe, Africa, North America and the other regions of Asia (more details see Supplementary Fig. S2). In addition to China in East Asia, some countries in Southeast Asia, mainly Vietnam and Indonesia, also maintained a great antigenic diversity since 2003 based on the HA-sequenced viruses. However, the composition and dominance of the antigenic types varies between countries. Taking 2006 as an example, AH05 dominated in China, while VN04 was dominant in Vietnam and Thailand, and ID05 in Indonesia; other countries reported yet other dominant ACs. Thus, antigenic differences between countries/regions further contributed to the overall antigenic diversity in East and Southeast Asia. Co-circulation of multiple ACs coincided with local persistence of some ACs. For example, ID11 was only detectable in HA-sequenced viruses in Indonesia, where it was detected over multiple years (Fig. 5a and Supplementary Fig. S2).

At a global scale, the co-circulation of various ACs is even more evident. Figure 5b summarizes the global composition of ACs in each year between 2003 and 2015. As a word of warning, the relative ratios displayed here are based on the HA-sequenced isolates, which may not reflect their true ratios due to sampling bias. Nevertheless, co-circulation of up to 11 ACs throughout the globe per year was observed since 2003 (detailed information regarding circulation of these ACs in different countries/regions over time is shown in Supplementary Figs S2 and S3).

Finally, for comparison we analysed antigenic clusters of human influenza H3N2 viruses (Fig. 5c), which does not show the large antigenic complexity of avian H5N1. For the human influenza H3N2 virus, there were typically one or two ACs circulating across the globe per year. The replacement of dominant ACs in the global population of human influenza H3N2 viruses in successive periods of approximately 3 to 4 years is also obvious.

## Discussion

In this study antigenic modeling was performed, based on existing HA1 sequences of HPAI H5N1 viruses and supplemented with 218 newly sequenced strains isolated during 2004–2013 from mainland China. This analysis provides a comprehensive picture of the antigenic evolution of HPAI H5N1 virus across the globe. We not only tracked the origins of different antigenic ACs, but also found that generation of antigenic diversity came with the spread of the virus in China. The continuous antigenic divergence of the virus has lead to extensive co-circulation and local persistence of ACs in recent years.

The antigenic evolution of human influenza H3N2 and H1N1 viruses is reported to be in a cluster-wise and trunk-like pattern, i.e., it can be viewed as the serial replacement of one AC by another^13^,^30^. In contrast, the HPAI H5N1 virus seems to evolve according to a divergent pattern, whereby ACs can evolve in multiple directions, as visualized in Figs 2b and 3. New ACs of this strain seem to emerge at high frequencies, suggested by the emergence of 15 major ACs and 21 minor ACs from a single lineage since 1996, while for human H3N2 viruses only 7 ACs circulated during 1996–2009 (Fig. 5c). This makes a sharp contrast to the antigenic evolution of other avian and swine influenza viruses, such as avian H9N2^20^, H7^31^,^32^ and swine H3N2 viruses^33^, for which only a few antigenic clusters were observed and the rate of antigenic evolution is much lower than that of HPAI H5N1 viruses. The rapid generation of new ACs of the HPAI H5N1 virus imposes a larger challenge for HPAI H5N1 virus surveillance and vaccination strategies.

It has been suggested that the source of HPAI H5N1 virus was southern China^28^,^29^, and the first HPAI H5N1 virus of the GsGD lineage was indeed detected in Guangdong province of southern China in 1996^2^. Then, the ‘Qinghai’ virus^34^ and ‘Fujian’ virus^35^ also emerged in China. In this study, we produce a more detailed picture of the antigenic evolution of HPAI H5N1 virus in China (Fig. 4a). The result showed that the development of its antigenic diversity was co-incident with its geographic expansion in China. This may be caused in part by the continuing pressure imposed by vaccination across the country, since mass vaccination campaigns have been conducted as a routine measure for the control of avian influenza viruses in China since 2004^3^,^17^. Despite these efforts, the virus has caused epidemics nearly throughout the complete country.

Besides for China, Southeast Asia and Egypt are two major host-spot regions for epidemics caused by HPAI H5N1 viruses (Fig. 5a and Supplementary Fig. S2). Large amounts of poultry were raised in these regions^36^,^37^. Although large antigenic diversity was observed in these regions, most of the ACs circulating there were introduced from China^28^,^38^. Few or minor antigenic drifts were observed in most countries except in Vietnam^39^,^40^, Indonesia^41^ and Egypt^42^,^43^ where significant antigenic drift happened. It is noteworthy that, three novel ACs ID11, EG07 and EG08 were generated in Indonesia and Egypt, respectively. It is of great concern that these new ACs can cause a new round of epidemics if they move out of these countries. Therefore, it is important to strengthen the surveillance for HPAI H5N1 virus in these countries. In addition, new ACs may also be generated in the countries with dense human and poultry populations like Bangladesh and India^44^,^45^.

To control the HPAI H5N1 virus, vaccinations have been conducted worldwide, especially in China, Indonesia, Vietnam and Egypt, where more than 99% of avian influenza vaccines were used. As is reported in previous studies^19^,^20^,^21^, vaccination programs could induce faster rates of antigenic drift in avian influenza viruses, especially when there are antigenic differences between the vaccine strain and the epidemic viruses. The large antigenic diversity and frequent drifts in the above four countries suggested that vaccination may drive the antigenic evolution of this virus in these countries. To promote the efficiency of vaccination, better vaccination strategies should be adapted to match the vaccines with circulating strains. Systematic antigenic grouping of this virus could facilitate such a vaccination strategy. As is shown above, local persistence and co-circulation of ACs are widely observed for the virus, which suggests that the application of vaccines should be based on the epidemic ACs in a country/region. For countries or regions with multiple ACs co-circulating, such as China and Southeast Asia, multiple vaccines or the universal vaccine should be provided; while for most countries in Europe, Africa and Middle East where QH05 mainly circulated, the vaccine against QH05 should be enough for protection of infections by HPAI H5N1 virus.

Our study could be biased towards the sequence distribution by region. As shown in our study, most of the sequence data (70%) came from China (including Hong Kong), Vietnam, Indonesia and Egypt (Supplementary Fig. S4). This could reflect their hot spot roles in HPAI H5N1 dissemination, as is exemplified by the observation that all the major ACs originated in these four countries, but it could also be a reflection of less thorough surveillance and sequencing efforts in other countries. Anyway, the viruses in these four countries captured almost all the antigenic diversity (Supplementary Table S6), suggesting the importance of strengthening the surveillance in these countries.

Overall, the coupling of large-scale HA sequencing and high-accuracy antigenic modeling will be a valuable tool not only for systematic understanding of the antigenic evolution of influenza viruses, but also for timely surveillance of new ACs, which could help for vaccine recommendations for HPAI H5N1 prevention and control.

## Methods

### HA sequence data

HA sequencing of 218 HPAI H5N1 viruses sampled from diverse regions of mainland China between 2004 and 2013 (Supplementary Table S1) were carried out according to the methods described in Supplemental Information. Other HA sequences of HPAI H5N1 virus with length greater than 900 were collected from the EpiFlu database of Global Initiative on Sharing All Influenza Data (GISAID)^46^ on January 27, 2016. The acknowledgement table for these viruses was available at http://www.computationalbiology.cn/material/. Only the sequences of HPAI H5N1 viruses in GsGD lineage were kept. All the remaining sequences were aligned using the software MAFFT^47^ with an additional manual check. The non-coding regions and the region coding for signal peptide were removed for each sequence. After removing the re-assortment sequences synthesized in laboratory, the sequences with gap content greater than 10% and the sequences stopped abnormally when translated into protein, we obtained in total 5387 DNA sequences with each 960 nucleotides long. They were then translated into protein sequences. The software cd-hit^48^ was used to remove the redundant sequences of 100% similarity. Finally, 2441 unique protein sequences were obtained. The information of all the viruses used in this study was available at http://www.computationalbiology.cn/material/.

### Antigenic clustering based on RPEDAC-H5-C

The ACs were predicted with the computational method PREDAC-H5-C which was adapted from PREDAC, a computational method for prediction of ACs for the human influenza H3N2 virus^27^. It included four steps: firstly, the antigenic relationship between any pairs of viruses used in this study were predicted based on HA1 protein sequences with the computational method PREDAC-H5 developed in our previous study^49^,^50^; secondly, any pair of viruses which was predicted to be antigenically similar was connected by an edge, which resulted in the antigenic correlation network (ACnet); thirdly, the ACnet would be separated into clusters using the software MCL^51^; finally, an antigenic dataset composed of 798 pairs of HPAI H5N1 viruses with known antigenic relationship were used to help determine the best antigenic clustering (details see Supplementary Methods).

Based on PREDAC-H5-C, all the HPAI H5N1 viruses used in this study were grouped into 36 ACs in total (Supplementary Table S2). Among them, 15 ACs were considered as major ACs, which circulated in no less than three years and caused epidemics (ratio greater than 20%) in its dominant country (the country covering most viruses of the cluster) in at least one year. The major ACs included over 97% of the viruses. The remaining ACs were defined as minor ACs.

### ACnet visualization

The ACnet was visualized using the yFiles Organic layout in Cytoscape^52^. To display the network clearly in Fig. 2a, only the unique protein sequences described above and the connections between them were used (Fig. 2a). To better display predicted ACs, the positions of some nodes were manually adjusted so that each predicted major AC had a clear boundary from the others.

### Clade determination

The clade of each virus belongs to in the nomenclature system was determined with Highly Pathogenic H5N1 Clade Classification Tool on Influenza Research Database^53^, which is available at http://www.fludb.org/brc/h5n1Classifier.spg?method=ShowCleanInputPage&decorator=influenza.

### Phylogenetic reconstruction and bayesian coalescent analysis

To infer the phylogenetic relationship between the HPAI H5N1 viruses used in the ACnet, the DNA sequences of the viruses used in the network were used to determine the phylogenetic tree with the help of software MEGA 5.2^54^. The maximum likelihood method was used with the general reversible GTR+I+c4 model. The tree was rooted with the virus GsGD. The coloring and visualization of the tree were done with script colorTree.pl^55^ and Dendroscope^56^, respectively. The detailed phylogenetic tree file is available at http://www.computationalbiology.cn/material/.

The time and country for the MRCAs of major ACs were inferred by Bayesian MCMC sampling using the software BEAST v1.75^57^ with the SRD06 codon position model and the uncorrelated exponential clock model. To reduce the computational cost, the earliest 20 viruses in each AC were chosen for analysis after removing the redundancy by country and month. Bayesian MCMC sampling was run for up to 100,000,000 times to achieve convergence. The summary tree file is available at http://www.computationalbiology.cn/material/.

### HI experiment

The antigenic characterization of six representative viruses of four antigenic clusters which were mainly composed of viruses of clade 2.3.4 and its subclades (2.3.4.1~2.3.4.4), including A/Anhui/1/2005 (AH05), A/Chicken/Hong Kong/AP156/2008 (Minor-28), A/Guizhou/1/2013 (GZ13), A/duck/Hubei/Hangmei01/2006 (AH05), A/Environment/Chongqing/16/2011 (AH05) and A/Environment/Jiangxi/20983/2013 (JX13), were determined by HI assays according to standard protocols proposed by WHO^58^ with ferret antisera and 0.5% turkey red blood cells. The antigenic cartography was generated with Smith’s method^30^.

## Additional Information

**How to cite this article**: Peng, Y. *et al*. Continual Antigenic Diversification in China Leads to Global Antigenic Complexity of Avian Influenza H5N1 Viruses. *Sci. Rep.* **7**, 43566; doi: 10.1038/srep43566 (2017).

**Publisher's note:** Springer Nature remains neutral with regard to jurisdictional claims in published maps and institutional affiliations.

## Supplementary Material

Supplementary Information
