# Corrigendum: Continual Antigenic Diversification in China Leads to Global Antigenic Complexity of Avian Influenza H5N1 Viruses

**DOI:** 10.1038/srep46994

**Published:** 2018-05-31

**Authors:** Yousong Peng, Xiaodan Li, Hongbo Zhou, Aiping Wu, Libo Dong, Ye Zhang, Rongbao Gao, Hong Bo, Lei Yang, Dayan Wang, Xian Lin, Meilin Jin, Yuelong Shu, Taijiao Jiang

Scientific Reports 7: Article number: 4356610.1038/srep43566; published online: 03 06 2017; updated: 05 31 2018

This Article contains an error in Figure 2, where the antigenic cluster label ‘AH05’ is missing.

In addition, this Article contains an error in the legend of Figure 2,

“**(c)** The antigenic cartography for six representative viruses of four antigenic clusters which were mainly composed of viruses of clade 2.3.4 and its sub-clades. The viruses were colored according to the antigenic clusters they belong to.”

should read:

“**(c)** The antigenic cartography for six representative viruses of four antigenic clusters which were mainly composed of viruses of clade 2.3.4 and its sub-clades. The viruses were colored according to the antigenic clusters they belong to. A/Chick/HK/AP156/2008 refers to A/Chicken/Hong Kong/AP156/2008.”

The correct Figure 2 appears below as Figure 1.

Finally, this Article contains errors in Affiliation 1, which is incorrectly listed as “College of Biology, Human University, Changsha, 410082, China”. The correct affiliation is listed below:

College of Biology, Hunan University, Changsha, 410082, China

## Figures and Tables

**Figure 1:**
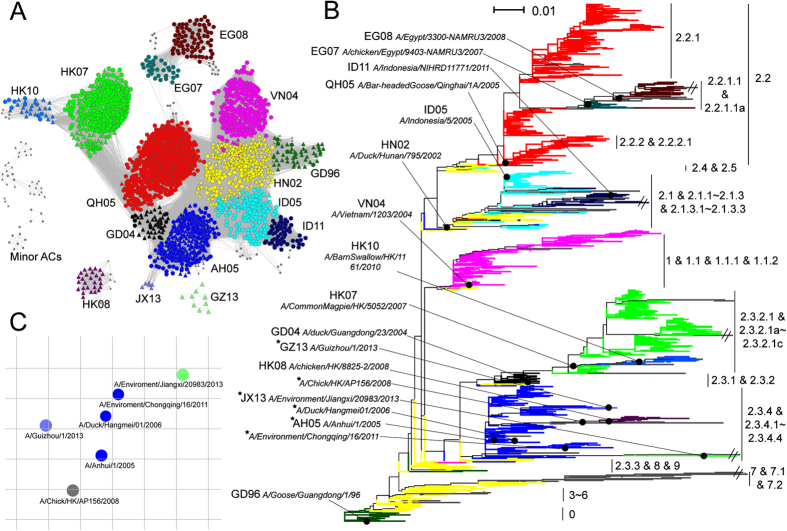
High-confidence modeling of ACs of HPAI H5N1 viruses. (**a**) Predicted antigenic correlation network (ACnet) of the ACs defined for 2441 HPAI H5N1 viruses with unique HA1 protein sequences. All pairs of viruses which were predicted to be antigenically similar were connected in ACnet. Triangles in the network refer to the viruses from China. The names for the major ACs (in color) are indicated, while minor ACs are shown in gray. (**b**) Phylogenetic tree of the 2441 HA1 sequences, colored according to the predicted ACs. The sub-clades to which the viruses belong (H5N1 Evolution Working Group nomenclature) are shown to the right. The branch length was scaled according to the legend in the top left. The strains listed to the left of the tree refer to the strains used for naming the predicted major ACs. The stars indicate strains used in the HI assay. (**c**) The antigenic cartography for six representative viruses of four antigenic clusters which were mainly composed of viruses of clade 2.3.4 and its sub-clades. The viruses were colored according to the antigenic clusters they belong to. A/Chick/HK/AP156/2008 refers to A/Chicken/Hong Kong/AP156/2008.

