# Supplementary material for: Continual Antigenic Diversification in China Leads to Global Antigenic Complexity of Avian Influenza H5N1 Viruses
Source: Sci Rep. 2017 Mar 6;7:43566. doi: 10.1038/srep43566 (PMC5337931; doi:10.1038/srep43566)
Supplement: Supplementary Information [file srep43566-s1.pdf]

Supplementary Information for

Continual Antigenic Diversification in China Leads to Global Antigenic Complexity of Avian Influenza H5N1 Viruses

Yousong Peng[1, 2, #], Xiaodan Li[3, #], Hongbo Zhou[4], Aiping Wu[5, 6], Libo Dong[3], Ye Zhang[3], Rongbao Gao[3], Hong Bo[3], Lei Yang[3], Dayan Wang[3], Xian Lin[4], Meilin Jin[4, *], Yuelong Shu[3, *], Taijiao Jiang[2, 5, 6, *]

[1] College of Biology, Human University, Changsha 410082, China

[2] Institute of Biophysics, Chinese Academy of Sciences, Beijing 100101, China

[3] National Institute for Viral Disease Control and Prevention, China CDC, Beijing 100052, China

[4] College of Animal Science & Medicine, Huazhong Agricultural University, Wuhan 430070, China

[5] Center of System Medicine, Institute of Basic Medical Sciences, Chinese Academy of Medical Sciences & Peking Union Medical College, Beijing, 100005, China

[6] Suzhou Institute of Systems Medicine, Suzhou, Jiangsu, 215123, China

[#] These authors contributed equally to this work

* Corresponding author: ML, +86-027-87286905, jml8328@126.com; YS, +86-010-58900850, yshu@cnic.org.cn; TJ, +86- 010-64888427, taijiao@ibms.pumc.edu.cn

Supplementary Methods

Isolation of the virus. The viral samples obtained from poultry markets were maintained in a viral-transport medium at −80° C until propagation. The specimens were propagated in the allantoic sac cavity of 9~11 days-old specific pathogen-free embryonated chicken eggs for 48 to 72 hours at 37° C.

RNA extraction and RT-PCR. RNA was extracted from virus isolates with the use of the QIAamp Viral RNA Mini Kit (Qiagen), according to the manufacturer's instructions. RT-PCR assays with specific primers for detecting H5N1 subtype were performed according to the protocols proposed by WHO [1].

Gene sequencing. Full length HA gene was amplified for sequencing, with the use of Qiagen OneStep RT-PCR Kit. PCR products were purified from agarose gel with the use of the QIA quick Gel Extraction Kit (Qiagen). We performed the sequencing using an ABI 3730xl automatic DNA analyzer (Life Technologies) and the ABI Big Dye Terminator v3.1 cycle sequencing kit (Life Technologies), according to the manufacturers' recommendations. Full length HA sequences of the viruses were deposited in the Global Initiative on Sharing Avian Influenza Data

(GISAID) database [2] (Table S1).

Determine the best antigenic clustering with a HI dataset

In MCL clustering, the inflation value is the only parameter which affects the cluster granularity in the MCL program[3]. It is usually chosen somewhere in a range: a bigger value will tend to result in fine-grained clusterings, and a smaller value will tend to result in very coarse grained clusterings. To determine the best inflation value under which the antigenic clustering could reflect the actual antigenic evolution of HPAI H5N1 viruses as accurate as possible, an antigenic dataset including 798 pairs of viruses with known antigenic relationship was constructed (available at http://www.computationalbiology.cn/material/). None of the pairs were used in the training of PREDAC-H5-C. All the viruses within the dataset were included in the antigenic clustering. They were grouped into ACs. The idea here is that if the antigenic clustering accurately captures the actual antigenic relationship of HPAI H5N1 viruses, the viruses within the predicted ACs should tend to be antigenically similar, while those between the predicted ACs should tend to be antigenically different. Therefore, we calculated the ratio of viral pairs which are both antigenic different and between ACs, or both antigenic similar and within ACs. The antigenic clustering with the highest ratio was chosen (Fig. S1).

Supplementary Figures

Figure S1: the agreement rate between the antigenic data and the clustering versus the inflation value used in the clustering by MCL. The arrow refers to the inflation value (1.7) under which the final antigenic clustering was derived by MCL for further analysis in the manuscript.

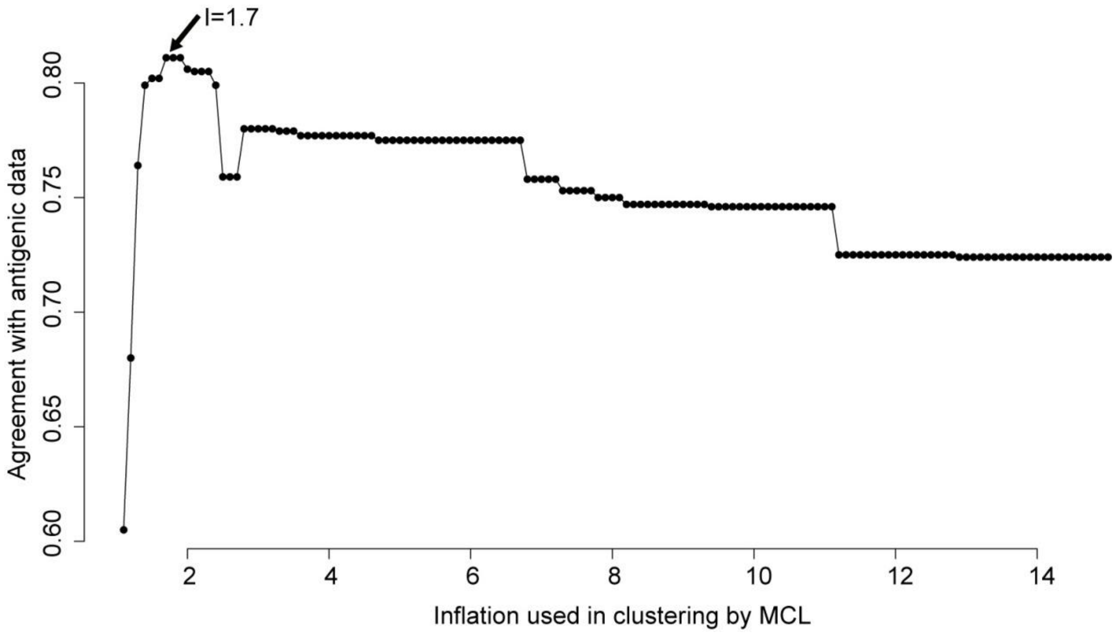

Figure S2. The spatiotemporal dynamics of the ACs in the globe from 1996 to 2015. Dynamic changes in the percentage of ACs were recorded on a yearly basis for the each country. The percentage of ACs in a year was in proportion to the size of the colored rectangle in the year. The white refers to no virus in the province in the given year.

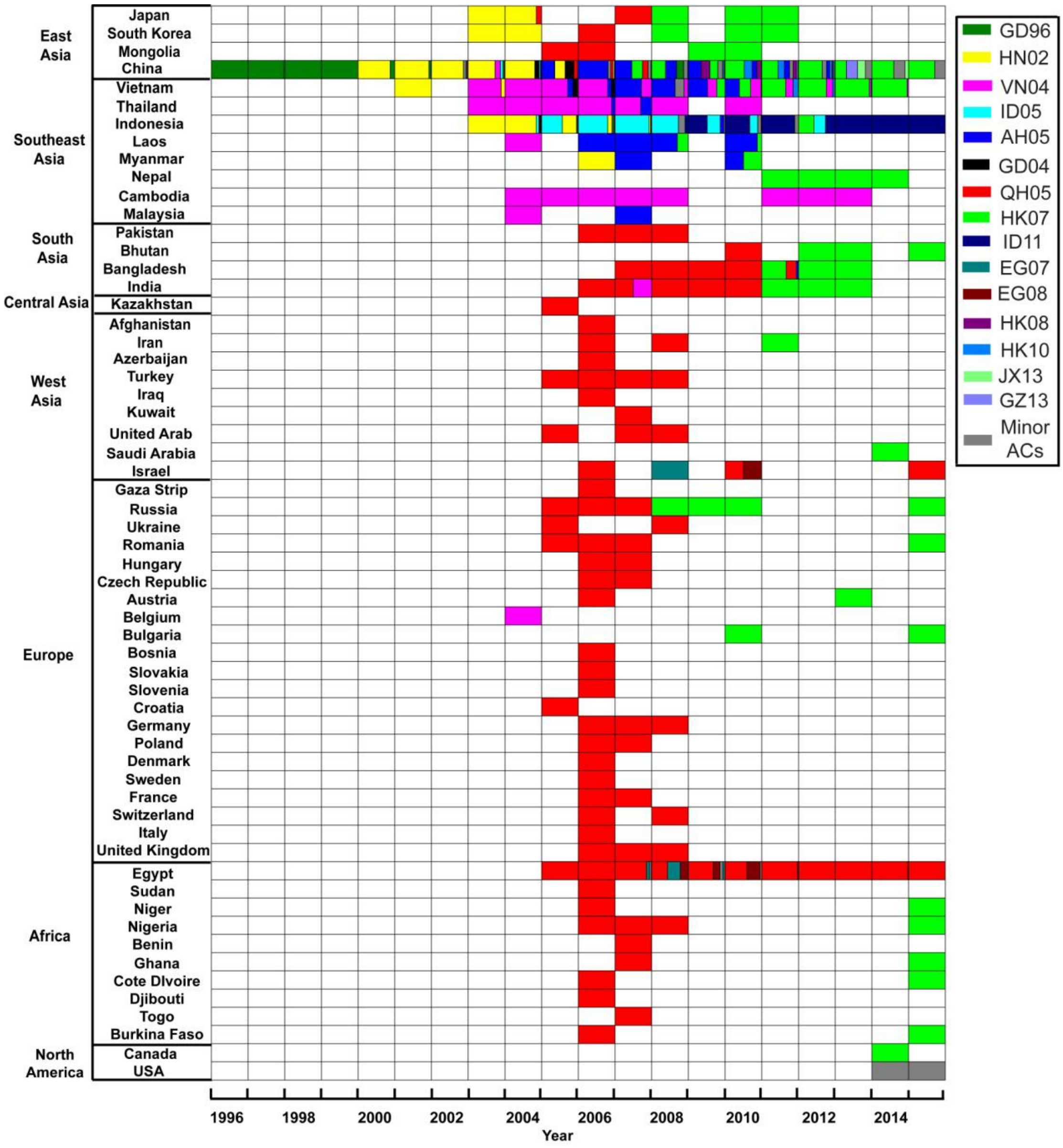

Figure S3. The number of co-circulating ACs each year in the globe from 1996 to 2015. The dashed line refers to the average yearly number of co-circulating ACs during this period.

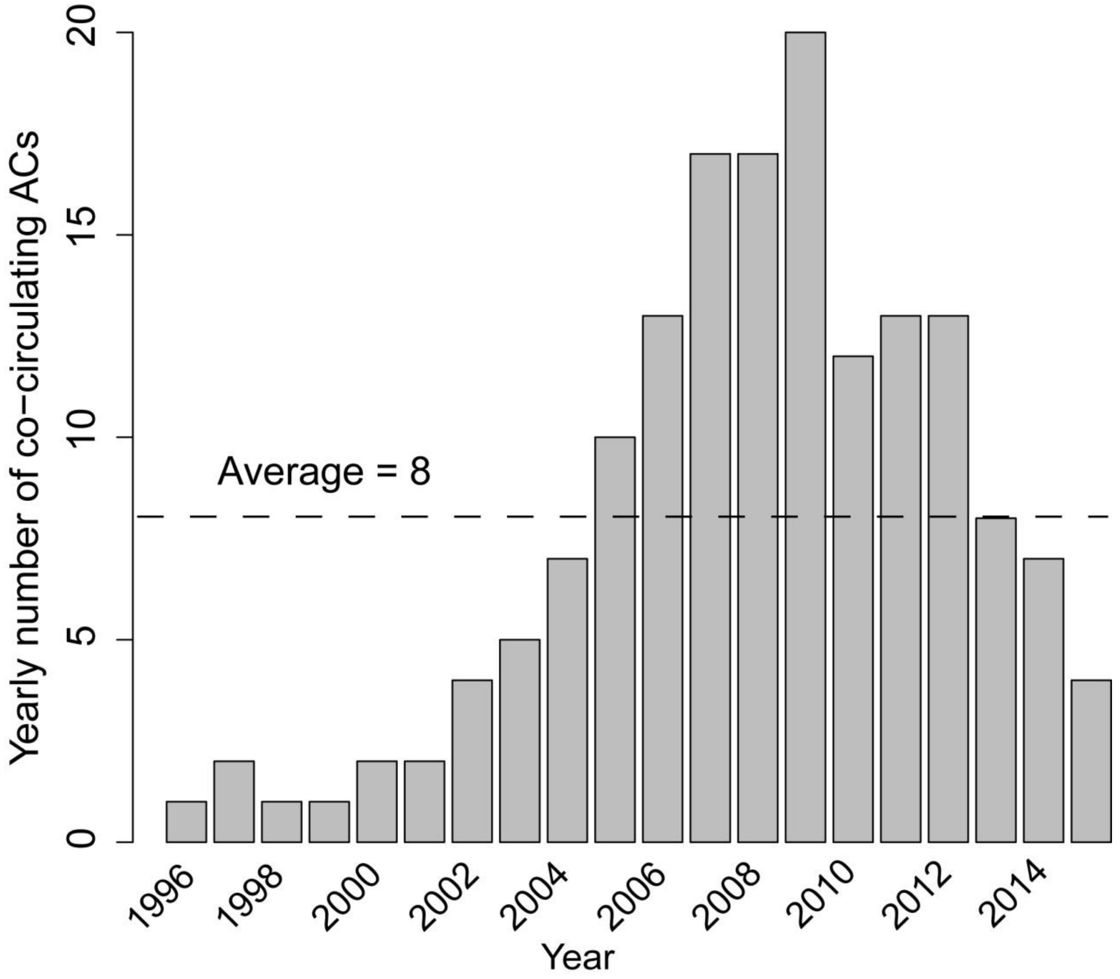

Figure S4. The country composition of the HPAI H5N1 viruses used in this study.

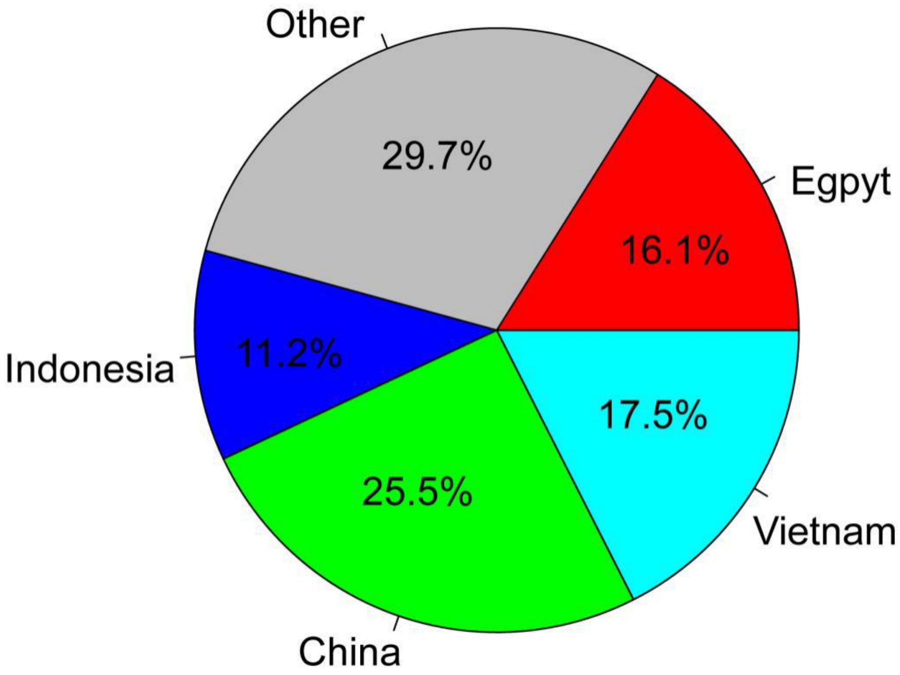

Supplementary Tables

Table S1. Sequencing of 218 viruses in mainland China used in this study. The newly sequenced viruses included 36 viruses from poultry (chicken, duck, goose and pigeon), two viruses from wild birds (widgeon and heron), 14 viruses from swine and 166 viruses from environment.

Isolate_Name	Isolate_Id	HA Segment_Id	Collection_Date
A/Chiken/HuBei/14a/2004	EPI_ISL_180527	EPI593068	2004-12-30
A/Duck/HuBei/Cg/2004	EPI_ISL_180529	EPI593070	2004-12-30
A/Chiken/HuBei/41/2004	EPI_ISL_179705	EPI591023	2004-12-30
A/Widgeon/HuBei/01/2004	EPI_ISL_180571	EPI593112	2004-12-30
A/Chiken/HuBei/ZF/2004	EPI_ISL_180528	EPI593069	2004-12-30
A/Chiken/HuNan/01/2006	EPI_ISL_180530	EPI593071	2006-01-10
A/Chiken/HuNan/02/2006	EPI_ISL_180531	EPI593072	2006-02-28
A/Swine/HuNan/01/2006	EPI_ISL_180532	EPI593073	2006-07-15
A/Swine/jiangXi/01/2006	EPI_ISL_180533	EPI593074	2006-08-22
A/Chiken/HuBei/01/2006	EPI_ISL_180534	EPI593075	2006-09-15
A/Duck/HuNan/02/2006	EPI_ISL_180535	EPI593076	2006-09-28
A/Duck/HuNan/03/2006	EPI_ISL_180537	EPI593078	2006-12-15
A/Chiken/HuNan/03/2006	EPI_ISL_180536	EPI593077	2006-12-15
A/Goose/HuNan/01/2006	EPI_ISL_180538	EPI593079	2006-12-19
A/Chiken/HuNan/04/2006	EPI_ISL_180539	EPI593080	2006-12-22
A/Duck/HuBei/01/2007	EPI_ISL_180540	EPI593081	2007-04-25
A/Duck/HuBei/02/2007	EPI_ISL_180541	EPI593082	2007-11-14
A/Chiken/HuBei/06/2007	EPI_ISL_180542	EPI593083	2007-11-25
A/Chiken/HuBei/12/2007	EPI_ISL_180545	EPI593086	2007-12-01
A/Pigeon/HuBei/01_1/2008	EPI_ISL_180572	EPI593113	2007-12-01
A/Chiken/HuBei/11/2007	EPI_ISL_180544	EPI593085	2007-12-01
A/Chiken/HuBei/10/2007	EPI_ISL_180543	EPI593084	2007-12-01
A/Duck/HuBei/06/2007	EPI_ISL_180546	EPI593087	2007-12-15
A/Pigeon/HuBei/01_2/2008	EPI_ISL_180574	EPI593115	2008-01-15
A/Heron/HuBei/01/2007	EPI_ISL_180573	EPI593114	2008-01-20
A/Swine/HuBei/03/2008	EPI_ISL_180547	EPI593088	2008-08-01

A/Swine/HuBei/07/2008	EPI_ISL_180551	EPI593092	2008-10-14
A/Swine/HuBei/10-2/2008	EPI_ISL_180575	EPI593116	2008-10-14
A/Swine/HuBei/05/2008	EPI_ISL_180549	EPI593090	2008-10-14
A/Swine/HuBei/08/2008	EPI_ISL_180552	EPI593093	2008-10-14
A/Swine/HuBei/04/2008	EPI_ISL_180548	EPI593089	2008-10-14
A/Swine/HuBei/11/2008	EPI_ISL_180554	EPI593095	2008-10-14
A/Swine/HuBei/06/2008	EPI_ISL_180550	EPI593091	2008-10-14
A/Swine/HuBei/09/2008	EPI_ISL_180553	EPI593094	2008-10-14
A/Chiken/HuBei/12/2008	EPI_ISL_180555	EPI593096	2008-12-22
A/environment/ZheJiang/8/2009	EPI_ISL_180525	EPI593066	2009-01-01
A/environment/ZheJiang/11/2009	EPI_ISL_180519	EPI593060	2009-01-01
A/environment/ZheJiang/4/2009	EPI_ISL_180524	EPI593065	2009-01-01
A/environment/ZheJiang/9/2009	EPI_ISL_180526	EPI593067	2009-01-01
A/Swine/HuBei/02/2009	EPI_ISL_180556	EPI593097	2009-01-30
A/Duck/HuBei/08/2009	EPI_ISL_180563	EPI593104	2009-02-13
A/Chiken/ShanDong/01/2009	EPI_ISL_180562	EPI593103	2009-02-14
A/environment/Jiangxi/8/2009	EPI_ISL_180505	EPI593046	2009-02-26
A/environment/Jiangxi/13/2009	EPI_ISL_180491	EPI593032	2009-02-26
A/environment/Jiangxi/10/2009	EPI_ISL_180488	EPI593029	2009-02-26
A/environment/Jiangxi/5/2009	EPI_ISL_180502	EPI593043	2009-02-26
A/environment/Jiangxi/3/2009	EPI_ISL_180500	EPI593041	2009-02-26
A/environment/Jiangxi/7/2009	EPI_ISL_180504	EPI593045	2009-02-26
A/environment/Jiangxi/2/2009	EPI_ISL_180494	EPI593035	2009-02-26
A/environment/Jiangxi/6/2009	EPI_ISL_180503	EPI593044	2009-02-26
A/environment/Jiangxi/4/2009	EPI_ISL_180501	EPI593042	2009-02-26
A/environment/Jiangxi/1/2009	EPI_ISL_180487	EPI593028	2009-02-26
A/environment/Jiangxi/12/2009	EPI_ISL_180490	EPI593031	2009-02-26
A/environment/Jiangxi/9/2009	EPI_ISL_180506	EPI593047	2009-02-26
A/environment/Jiangxi/11/2009	EPI_ISL_180489	EPI593030	2009-02-26
A/environment/ZheJiang/2/2009	EPI_ISL_180523	EPI593064	2009-03-03
A/environment/ZheJiang/1/2009	EPI_ISL_180518	EPI593059	2009-03-03
A/environment/JiangXi/22/2009	EPI_ISL_180497	EPI593038	2009-04-17
A/environment/JiangXi/23/2009	EPI_ISL_180498	EPI593039	2009-04-17
A/environment/JiangXi/21/2009	EPI_ISL_180496	EPI593037	2009-04-17
A/Swine/HuBei/06-2/2009	EPI_ISL_180576	EPI593117	2009-05-24
A/Duck/HuBei/03/2009	EPI_ISL_180558	EPI593099	2009-07-09
A/Duck/HuBei/04/2009	EPI_ISL_180559	EPI593100	2009-07-09
A/Duck/HuBei/13/2009	EPI_ISL_180560	EPI593101	2009-07-09
A/Duck/HuBei/02/2009	EPI_ISL_180557	EPI593098	2009-07-09
A/swine/SiChuan/1/2009	EPI_ISL_180564	EPI593105	2009-08-03
A/Chiken/HuBei/07/2009	EPI_ISL_180561	EPI593102	2009-08-05
A/environment/WuHan/03/2009	EPI_ISL_180512	EPI593053	2009-12-02
A/environment/Anhui/05/2010	EPI_ISL_180363	EPI592904	2009-12-29

A/environment/Fujian/02/2010	EPI_ISL_180435	EPI592976	2010-01-19
A/environment/Fujian/32/2010	EPI_ISL_180454	EPI592995	2010-01-19
A/environment/Fujian/06/2010	EPI_ISL_180438	EPI592979	2010-02-08
A/environment/Fujian/04/2010	EPI_ISL_180436	EPI592977	2010-02-08
A/environment/Fujian/05/2010	EPI_ISL_180437	EPI592978	2010-02-08
A/environment/Fujian/07/2010	EPI_ISL_180439	EPI592980	2010-02-08
A/environment/Chongqing/37/2010	EPI_ISL_180422	EPI592963	2010-02-09
A/environment/Chongqing/36/2010	EPI_ISL_180421	EPI592962	2010-02-09
A/environment/Chongqing/6/2010	EPI_ISL_180431	EPI592972	2010-02-22
A/environment/Chongqing/8/2010	EPI_ISL_180433	EPI592974	2010-02-22
A/environment/Chongqing/7/2010	EPI_ISL_180432	EPI592973	2010-02-22
A/environment/Chongqing/5/2010	EPI_ISL_180426	EPI592967	2010-02-22
A/environment/Fujian/36/2010	EPI_ISL_180455	EPI592996	2010-03-15
A/environment/Fujian/16/2010	EPI_ISL_180447	EPI592988	2010-03-15
A/environment/Fujian/37/2010	EPI_ISL_180456	EPI592997	2010-03-17
A/environment/Fujian/12/2010	EPI_ISL_180444	EPI592985	2010-03-17
A/environment/Fujian/40/2010	EPI_ISL_180458	EPI592999	2010-03-17
A/environment/Fujian/10/2010	EPI_ISL_180442	EPI592983	2010-03-17
A/environment/Fujian/15/2010	EPI_ISL_180446	EPI592987	2010-03-17
A/environment/Fujian/41/2010	EPI_ISL_180459	EPI593000	2010-03-17
A/environment/Fujian/14/2010	EPI_ISL_180445	EPI592986	2010-03-17
A/environment/Fujian/39/2010	EPI_ISL_180457	EPI592998	2010-03-17
A/environment/Fujian/11/2010	EPI_ISL_180443	EPI592984	2010-03-17
A/environment/Chongqing/9/2010	EPI_ISL_180434	EPI592975	2010-03-23
A/environment/Chongqing/16/2010	EPI_ISL_180402	EPI592943	2010-03-23
A/environment/Chongqing/14/2010	EPI_ISL_180400	EPI592941	2010-03-23
A/environment/Chongqing/23/2010	EPI_ISL_180416	EPI592957	2010-03-23
A/environment/Chongqing/41/2010	EPI_ISL_180423	EPI592964	2010-03-25
A/Duck/HuBei/06/2010	EPI_ISL_180568	EPI593109	2010-03-28
A/Duck/HuBei/01/2010	EPI_ISL_180565	EPI593106	2010-03-28
A/Duck/HuBei/05/2010	EPI_ISL_180567	EPI593108	2010-03-28
A/Chiken/HuNan/01/2010	EPI_ISL_180566	EPI593107	2010-03-28
A/environment/Jiangxi/14/2011	EPI_ISL_180492	EPI593033	2010-03-29
A/environment/Chongqing/51/2010	EPI_ISL_180427	EPI592968	2010-03-31
A/environment/Guizhou/01/2010	EPI_ISL_180473	EPI593014	2010-04-01
A/environment/FuJian/21/2010	EPI_ISL_180450	EPI592991	2010-04-11
A/environment/Fujian/20/2010	EPI_ISL_180449	EPI592990	2010-04-11
A/environment/Fujian/26/2010	EPI_ISL_180453	EPI592994	2010-04-12
A/environment/Fujian/18/2010	EPI_ISL_180448	EPI592989	2010-04-15
A/environment/Chongqing/21/2010	EPI_ISL_180412	EPI592953	2010-04-21
A/environment/Chongqing/12/2010	EPI_ISL_180399	EPI592940	2010-04-21
A/environment/Chongqing/17/2010	EPI_ISL_180404	EPI592945	2010-04-21
A/environment/Hunan/01/2010	EPI_ISL_180480	EPI593021	2010-04-21

A/environment/Chongqing/19/2010	EPI_ISL_180408	EPI592949	2010-04-21
A/environment/Chongqing/18/2010	EPI_ISL_180406	EPI592947	2010-04-21
A/environment/Chongqing/20/2010	EPI_ISL_180410	EPI592951	2010-04-21
A/environment/Chongqing/42/2010	EPI_ISL_180424	EPI592965	2010-04-30
A/environment/Chongqing/43/2010	EPI_ISL_180425	EPI592966	2010-04-30
A/environment/Chongqing/55/2010	EPI_ISL_180428	EPI592969	2010-05-19
A/environment/Chongqing/56/2010	EPI_ISL_180429	EPI592970	2010-05-19
A/Duck/HuBei/09/2010	EPI_ISL_180570	EPI593111	2010-07-10
A/Duck/HuBei/08/2010	EPI_ISL_180569	EPI593110	2010-07-10
A/environment/Guizhou/02/2010	EPI_ISL_180474	EPI593015	2010-08-01
A/environment/Guizhou/06/2010	EPI_ISL_180477	EPI593018	2010-08-20
A/environment/Guizhou/03/2010	EPI_ISL_180475	EPI593016	2010-08-20
A/Duck/HuBei/10-2/2010	EPI_ISL_180577	EPI593118	2010-08-20
A/environment/Chongqing/22/2010	EPI_ISL_180414	EPI592955	2010-09-21
A/environment/Chongqing/24/2010	EPI_ISL_180418	EPI592959	2010-09-21
A/environment/Fujian/22/2010	EPI_ISL_180451	EPI592992	2010-10-12
A/environment/Guangdong/01/2010	EPI_ISL_180461	EPI593002	2010-11-15
A/environment/Chongqing/57/2010	EPI_ISL_180430	EPI592971	2010-11-15
A/environment/FuJian/24/2010	EPI_ISL_180452	EPI592993	2010-11-16
A/environment/Guizhou/05/2010	EPI_ISL_180476	EPI593017	2010-12-01
A/environment/Chongqing/29/2010	EPI_ISL_180420	EPI592961	2010-12-08
A/environment/Chongqing/26/2010	EPI_ISL_180361	EPI592902	2010-12-08
A/environment/Zhejiang/12/2010	EPI_ISL_180521	EPI593062	2010-12-13
A/environment/ZheJiang/11/2010	EPI_ISL_180520	EPI593061	2010-12-13
A/environment/Guangxi/03/2010	EPI_ISL_180466	EPI593007	2010-12-17
A/environment/Zhejiang/13/2010	EPI_ISL_180522	EPI593063	2010-12-29
A/environment/Hunan/05/2011	EPI_ISL_180482	EPI593023	2011-01-18
A/environment/Hunan/03/2011	EPI_ISL_180481	EPI593022	2011-01-18
A/environment/Chongqing/10/2011	EPI_ISL_180397	EPI592938	2011-01-24
A/environment/Chongqing/11/2011	EPI_ISL_180398	EPI592939	2011-01-24
A/environment/Chongqing/09/2011	EPI_ISL_180396	EPI592937	2011-01-24
A/environment/Chongqing/23/2011	EPI_ISL_180417	EPI592958	2011-01-25
A/environment/Chongqing/24/2011	EPI_ISL_180419	EPI592960	2011-01-25
A/environment/Xinjiang/01/2011	EPI_ISL_180513	EPI593054	2011-01-26
A/environment/Xinjiang/02/2011	EPI_ISL_180514	EPI593055	2011-01-26
A/environment/Hunan/07/2011	EPI_ISL_180483	EPI593024	2011-01-26
A/environment/Chongqing/07/2011	EPI_ISL_180394	EPI592935	2011-02-22
A/environment/Chongqing/06/2011	EPI_ISL_180393	EPI592934	2011-02-22
A/environment/Chongqing/03/2011	EPI_ISL_180390	EPI592931	2011-03-22
A/environment/Chongqing/05/2011	EPI_ISL_180392	EPI592933	2011-03-22
A/environment/Chongqing/22/2011	EPI_ISL_180415	EPI592956	2011-03-22
A/environment/Chongqing/04/2011	EPI_ISL_180391	EPI592932	2011-03-22
A/environment/Jiangxi/16/2011	EPI_ISL_180493	EPI593034	2011-03-24

A/environment/Chongqing/01908/2011	EPI_ISL_180370	EPI592911	2011-04-12
A/environment/Chongqing/01903/2011	EPI_ISL_180368	EPI592909	2011-04-12
A/environment/Chongqing/01904/2011	EPI_ISL_180369	EPI592910	2011-04-12
A/environment/Sichuan/01/2011	EPI_ISL_180510	EPI593051	2011-04-14
A/environment/Chongqing/21/2011	EPI_ISL_180413	EPI592954	2011-04-28
A/environment/Xinjiang/03/2011	EPI_ISL_180516	EPI593057	2011-04-28
A/environment/Chongqing/01891/2011	EPI_ISL_180367	EPI592908	2011-05-26
A/environment/Chongqing/16/2011	EPI_ISL_180403	EPI592944	2011-05-26
A/environment/Chongqing/19/2011	EPI_ISL_180409	EPI592950	2011-05-26
A/environment/Chongqing/20/2011	EPI_ISL_180411	EPI592952	2011-05-26
A/environment/Chongqing/18/2011	EPI_ISL_180407	EPI592948	2011-05-26
A/environment/Chongqing/17/2011	EPI_ISL_180405	EPI592946	2011-05-26
A/environment/Shaanxi/02011/2012	EPI_ISL_180508	EPI593049	2011-06-22
A/environment/Shaanxi/04/2011	EPI_ISL_180509	EPI593050	2011-07-03
A/environment/Shaanxi/01/2011	EPI_ISL_180507	EPI593048	2011-07-03
A/environment/Chongqing/08/2011	EPI_ISL_180395	EPI592936	2011-07-28
A/environment/Gansu/02071/2011	EPI_ISL_180460	EPI593001	2011-08-25
A/environment/Chongqing/15/2011	EPI_ISL_180401	EPI592942	2011-10-18
A/environment/Wuhan/02/2011	EPI_ISL_180511	EPI593052	2011-11-09
A/Environment/Xizang/07518/2011	EPI_ISL_180517	EPI593058	2011-12-25
A/environment/Guangdong/01277/2012	EPI_ISL_180463	EPI593004	2012-01-09
A/environment/Guangdong/01271/2012	EPI_ISL_180462	EPI593003	2012-01-09
A/environment/Chongqing/02418/2012	EPI_ISL_180375	EPI592916	2012-01-11
A/environment/Chongqing/02393/2011	EPI_ISL_180371	EPI592912	2012-01-13
A/environment/Chongqing/02412/2012	EPI_ISL_180373	EPI592914	2012-02-09
A/environment/Chongqing/02415/2012	EPI_ISL_180374	EPI592915	2012-02-09
A/environment/Chongqing/02409/2012	EPI_ISL_180372	EPI592913	2012-02-09
A/environment/Jiangxi/02128/2012	EPI_ISL_180485	EPI593026	2012-02-22
A/environment/Jiangxi/02165/2012	EPI_ISL_180486	EPI593027	2012-04-10
A/environment/Chongqing/02454/2012	EPI_ISL_180380	EPI592921	2012-04-16
A/environment/Chongqing/02455/2012	EPI_ISL_180381	EPI592922	2012-04-16
A/environment/Chongqing/02449/2012	EPI_ISL_180378	EPI592919	2012-04-16
A/environment/Chongqing/02450/2012	EPI_ISL_180379	EPI592920	2012-04-16
A/environment/Chongqing/024362012	EPI_ISL_180377	EPI592918	2012-04-20
A/environment/Chongqing/02433/2012	EPI_ISL_180376	EPI592917	2012-05-18
A/environment/Hunan/00245/2013	EPI_ISL_180479	EPI593020	2012-07-04
A/environment/Chongqing/02648/2011	EPI_ISL_180382	EPI592923	2012-07-12
A/environment/Chongqing/02933/2012	EPI_ISL_180388	EPI592929	2012-07-18
A/environment/Xinjiang/02678/2012	EPI_ISL_180515	EPI593056	2012-07-25
A/environment/Chongqing/02917/2012	EPI_ISL_180384	EPI592925	2012-08-16
A/environment/Chongqing/02916/2012	EPI_ISL_180383	EPI592924	2012-08-16
A/environment/Chongqing/02920/2012	EPI_ISL_180385	EPI592926	2012-08-17
A/environment/Chongqing/02934/2012	EPI_ISL_180389	EPI592930	2012-09-14

A/environment/Chongqing/02927/2012	EPI_ISL_180386	EPI592927	2012-10-16
A/environment/Chongqing/02930/2012	EPI_ISL_180387	EPI592928	2012-10-16
A/environment/Guangxi/00131/2013	EPI_ISL_180465	EPI593006	2012-12-05
A/environment/Guizhou/00320/2013	EPI_ISL_180472	EPI593013	2013-02-10
A/environment/Guizhou/00318/2013	EPI_ISL_180471	EPI593012	2013-02-11
A/environment/Guizhou/00314/2013	EPI_ISL_180470	EPI593011	2013-02-11
A/environment/Guizhou/00312/2013	EPI_ISL_180469	EPI593010	2013-02-13
A/environment/Guizhou/00311/2013	EPI_ISL_180468	EPI593009	2013-02-13
A/environment/Guizhou/00310/2013	EPI_ISL_180467	EPI593008	2013-02-13
A/environment/Chongqing/00410/2013	EPI_ISL_180365	EPI592906	2013-02-17
A/environment/Chongqing/00408/2013	EPI_ISL_180364	EPI592905	2013-02-18
A/Environment/Hubei/00950/2013	EPI_ISL_180478	EPI593019	2013-02-26
A/environment/Anhui/00464/2013	EPI_ISL_180362	EPI592903	2013-03-18
A/environment/Chongqing/00510/2013	EPI_ISL_180366	EPI592907	2013-03-19
A/environment/Jiangxi/00433/2013	EPI_ISL_180484	EPI593025	2013-03-21
A/Environment/Guangdong/07336/2013	EPI_ISL_180464	EPI593005	2013-10-14
A/Environment/Fujian/08415/2013	EPI_ISL_180441	EPI592982	2013-10-30
A/Environment/Fujian/08414/2013	EPI_ISL_180440	EPI592981	2013-10-30
A/Environment/Jiangxi/20983/2013	EPI_ISL_180495	EPI593036	2013-11-26
A/Environment/Jiangxi/23094/2013	EPI_ISL_180499	EPI593040	2013-12-14

Table S2. The detailed information about the ACs predicted by PREDAC-H5-C. The table shows the number of sequences in each AC, the time range and the clade composition of each AC. The major ACs are highlighted by the bold type; the remaining are the minor clusters. For clarity, only the clades with ratio greater than 1% are given in the table.

Cluster ID	Cluster Name	Sequence number	Year range	Clades composition
1	**QH05**	1570	2004-2015	2.2.1=0.511 2.2=0.397 2.2.2=0.037 2.2.2.1=0.034 2.2-like=0.019 2-like=0.001 2.2.1-like=0.001 2.5=0.001 2.1-like=0.001
2	**HK07**	861	2007-2015	2.3.2.1c=0.472 2.3.2.1a=0.387 2.3.2.1=0.136 2.3.2=0.005 2.3.2.1-like=0.001
3	**AH05**	840	2003-2013	2.3.4=0.595 2.3.4.3=0.212 2.3.4.2=0.098 2.3.4.1=0.074 2.1.3.3=0.007 2.3-like=0.007 2.1.3=0.001 9=0.001 2.3.2=0.001 2.3.4.4=0.001 1=0.001 2.3.1=0.001
4	**HN02**	476	2000-2009	3=0.118 0=0.113 9=0.107 2.1.1=0.107 2.1.2=0.086 7=0.067 2.3.3=0.063 1=0.057 5=0.055 2.4=0.038 2.3.1=0.036 2.5=0.029 6=0.019 2-like=0.019 1-2-like=0.015 1-2-5-6-8-9-like=0.015 5-6-like=0.013 2.1.3.1=0.011 2.1-like=0.011 4=0.011 8=0.008 1-2-9-like=0.002 2.3-like=0.002
5	**VN04**	706	2002-2014	1=0.71 1.1.2=0.12 1.1=0.091 1.1.1=0.064 1.1-like=0.013 0=0.001 9=0.001
6	**ID05**	386	1997-2012	2.1.3.2=0.751 2.1.3=0.104 2.1.3.3=0.06 2.1.3.1=0.044 2.4=0.021 2.1.1=0.018 7=0.003
7	**EG08**	123	2007-2014	2.2.1.1a=0.707 2.2.1.1=0.293
8	**ID11**	102	2006-2015	2.1.3.2a=0.931 2.1.3.2=0.069
9	**GD96**	89	1996-2008	0=0.989 1-2-5-6-8-9-like=0.011
10	**GD04**	102	2004-2007	2.3.2=0.951 2.1.1=0.049
11	**HK10**	56	2009-2012	2.3.2.1b=0.982 2.3.2.1-like=0.018
12	**EG07**	64	2007-2009	2.2.1.1=1
13	**HK08**	42	2008-2012	2.3.4=1

14	Minor-14	12	2009-2013	7.2=1
15	Minor-15	13	2009-2010	2.2.1.1=1
16	Minor-16	9	2005-2007	7=1
17	**JX13**	9	2009-2014	2.3.4.4=1
18	Minor-18	18	2002-2006	4=1
19	Minor-19	7	2009-2011	2.1.3.1=1
20	Minor-20	19	2008-2008	7.1=1
21	**GZ13**	10	2011-2013	2.3.4.2=1
22	Minor-22	7	2007-2009	2.2.1.1=1
23	Minor-23	6	2007-2007	2.2.1=1
24	Minor-24	5	2011-2012	2.3.2.1b=1
25	Minor-25	10	2006-2008	2.1.3.2=1
26	Minor-26	6	2008-2009	7.2=1
27	Minor-27	5	2007-2008	7.2=1
28	Minor-28	4	2007-2008	2.3.4=1
29	Minor-29	6	2009-2009	2.3.4=1
30	Minor-30	3	2008-2009	7.2=1
31	Minor-31	4	2007-2009	2.1.3.2=1
32	Minor-32	5	2014-2015	2.3.2.1c=1
33	Minor-33	2	2006-2006	2.5=1
34	Minor-34	3	2005-2006	2.3.4=1
35	Minor-35	2	2007-2012	2.3.4=1
36	Minor-36	2	2010-2010	2.1.3.2a=1

Table S3. The antigenic relationship between vaccine strains for clade 2.2, and its sub-clades 2.2.1 and 2.2.1.1. The elements in gray-shaded refers to the rAHM distance [4] (which indicate antigenic similarity when it is small than 4), or the rNHT distance [4] (marked by asterisk, which indicate antigenic similarity when it is small than 8). “S”, antigenic similar; “D”, antigenic different; “-“, no data available; WS244, A/WhooperSwan/Mongolia/244/2005; BHG05, A/Bar-headedGoose/Qinghai/1A/2005; IN33487, A/Chicken/India/Niv-33487/2006; EG2321, A/Egypt/2321-NAMRU3/2007; TK05, A/Turkey/Turkey/1/2005; EG3072, A/Egypt/N03072/2010; EG3300, A/Egypt/3300-NAMRU3/2008.

Vaccine	*Clade*	*WS244*	*BHG05*	*IN33487*	*EG2321*	*TK05*	*EG3072*	*EG3300*
WS244	*2.2*		*S*	*S*	D	*S*	-	*D*
BHG05	*2.2*	*1*		*S*	*S*	*S*	-	*D*
IN33487	*2.2*	*1*	*1**		*S*	*S*	-	-
EG2321	*2.2.1*	*4*	*2*	*4**		*D*	*S*	*D*
TK05	*2.2.1*	*1.4*	*2*	*2**	*4*		*S*	*D*
EG3072	*2.2.1*	-	-	-	*2.8*	*2*		*D*
EG3300	*2.2.1.1*	*61*	*25*	-	*144*	*196*	*362*	

Table S4. The HI assay between six representative viruses of four antigenic clusters which were mainly composed of viruses of clade 2.3.4 and its sub-clades. Titers in bold refer to the homogenous titers.

Test viruses	Antigenic clusters	Clade	Anti-sera (ferret)		
			Anhui05	HK/AP156/08	Guizhou/13
A/Anhui/1/2005 (IBCDC-RG6)	AH05	2.3.4	**160**	320	160
A/Chick/HK/AP156/2008 (SJ002)	Minor-28	2.3.4	<20	**640**	80
A/Guizhou/1/2013 (RG35)	GZ13	2.3.4.2	20	160	**320**
Duck/Hangmei/01/06	AH05	2.3.4	80	80	160
Enviroment/Chongqing/16/11	AH05	2.3.4	40	80	80
Enviroment/Jiangxi/20983/13	JX13	2.3.4.4	<20	20	<20

Table S5. The inferred time for the most recent common ancestor, the inferred most probable source country, the earliest and latest emergence time for the major ACs.

AC	Source Time (95% confidence level)	Earliest emergence time	Latest emergence time	Source country (probability)
GD96	1991-11-3 (1987-7-22 ~ 1994-11-1)	1996	2008-8-9	China (0.96)
HN02	1995-1-14 (1993-3-9 ~ 1996-5-13)	2000	2009-2-21	China (1)
VN04	1995-1-14 (1993-3-9 ~ 1996-5-13)	2002	2014-1-28	China (1)
ID05	1995-9-9 (1994-3-5 ~ 1996-8-15)	1997	2012	China (0.99)
AH05	2001-3-25 (2000-6-8 ~ 2001-12-14)	2003	2013-6-5	China (1)
GD04	2001-3-25 (2000-6-8 ~ 2001-12-14)	2004-6-29	2007-5-29	China (1)
QH05	2002-1-13 (2001-5-29 ~ 2002-8-1)	2004	2015-6-28	China (0.99)
HK07	2006-1-25 (2005-6-29 ~ 2006-8-9)	2007-5-13	2015-8-6	China (1)
ID11	2006-4-23 (2005-10-13 ~ 2006-10-4)	2006-10-14	2015-3-24	Indonesia (1)
EG07	2006-7-10 (2006-1-31 ~ 2006-12-5)	2007	2009-10-1	Egypt (1)
EG08	2007-2-25 (2006-10-9 ~ 2007-6-18)	2007-7-29	2014-11-1	Egypt (1)
HK08	2007-8-17 (2007-3-17 ~ 2007-12-6)	2008	2012-3-3	China (1)
HK10	2008-4-2 (2007-9-7 ~ 2008-9-30)	2009-5-5	2012-9-21	China (1)
JX13	2009-9-14 (2009-5-5 ~ 2009-12-10)	2009-12-10	2014-1-10	China (1)
GZ13	2010-8-24 (2009-12-31 ~ 2011-2-26)	2011-4-12	2013-2-13	China (1)

Table S6: The ratio of viruses isolated from four hot-spot countries (China, Indonesia, Vietnam and Egypt) for each major antigenic clusters (ACs).

AC	Ratio	AC	Ratio	AC	Ratio	AC	Ratio	AC	Ratio
AH05	0.91	GD04	1	HK07	0.53	HN02	0.97	JX13	1
EG07	0.98	GD96	1	HK08	1	ID05	1	QH05	0.47
EG08	0.99	GZ13	1	HK10	1	ID11	1	VN04	0.52

Supplementary References

1 WHO Global Influenza Surveillance Network. *Manual for the laboratory diagnosis and virological surveillance of influenza*. (2011).

2 Peter Bogner, I. C., David J. Lipman, Nancy J. Cox. A global initiative on sharing avian flu data. *Nature* **442** (2006).

3 Enright, A. J., Van Dongen, S. & Ouzounis, C. A. An efficient algorithm for large-scale detection of protein families. *Nucleic Acids Res* **30**, 1575-1584 (2002).

4 Ndifon, W., Dushoff, J. & Levin, S. A. On the use of hemagglutination-inhibition for influenza surveillance: surveillance data are predictive of influenza vaccine effectiveness. *Vaccine* **27**, 2447-2452, doi:10.1016/j.vaccine.2009.02.047 (2009).
